# Experimental Study on Durability Degradation of Geopolymer-Stabilized Soil under Sulfate Erosion

**DOI:** 10.3390/ma15155114

**Published:** 2022-07-22

**Authors:** Guanci Wang, Shanling Chen, Minmin Xia, Weilin Zhong, Xuegang Han, Biao Luo, Mohanad Muayad Sabri Sabri, Jiandong Huang

**Affiliations:** 1 Yueyang Road and Bridge Group Co., Ltd., Yueyang 414021, China; wangguanci01@sohu.com (G.W.); zhongwl123@163.com (W.Z.); hxg930171@foxmail.com (X.H.); 2 Department of Transportation of Hunan Province, Traffic Manufacturing Cost Management Station, Changsha 410116, China; chenshanling001@sohu.com; 3 Zoomlion Heavy Industry Science & Technology Co., Ltd., Changsha 410013, China; xia.minmin@163.com; 4 College of Civil Engineering, Xiangtan University, Xiangtan 411105, China; 5 Peter the Great St. Petersburg Polytechnic University, St. Petersburg 195251, Russia; mohanad.m.sabri@gmail.com; 6 School of Civil Engineering, Guangzhou University, Guangzhou 510006, China

**Keywords:** geopolymer-stabilized soil, sulfate erosion, durability degradation, strength development, microstructure

## Abstract

In this study, the potential application of slag-fly ash-based geopolymers as stabilizers for soft soil in sulfate erosion areas was investigated to promote environmental protection and waste residue recycling. The changes in the physical and mechanical properties and microstructure characteristics of cement-stabilized soil/geopolymer-stabilized soil under sulfate erosion were comparatively studied through tests such as appearance change, mass change, strength development, and microscopic examination. The results show that the sulfate resistance of stabilized soil is significantly affected by the stabilizer type. In the sulfate environment, the cement-stabilized soil significantly deteriorates with erosion age due to the expansion stress induced by AFt, while the geopolymer-stabilized soil exhibits excellent sulfate resistance. The slag-fly ash ratio (10:0, 9:1, 8:2 and 7:3) is an important factor affecting the sulfate resistance of geopolymer-stabilized soils, and the preferred value occurs at 9:1 (G-2). When immersed for 90 d, the unconfined compressive strength value of G-2 is 7.13 MPa, and its strength retention coefficient is 86.6%. The N-A-S-H gel formed by the polymerization in the geopolymer contributes to hindering the intrusion of sulfate ions, thereby improving the sulfate resistance of stabilized soil. The research results can provide a reference for technology that stabilizes soil with industrial waste in sulfate erosion areas.

## 1. Introduction

Defects such as differential settlement, lateral spreading, and instability occur due to the low-strength, high-compressibility, and inhomogeneity of the natural soft soil subgrade under traffic loading, thus affecting the quality of highway construction and the safety of vehicle operation [1,2,3]. Chemical stabilization reinforcement is an effective method to solve the insufficient bearing performance of the in situ soft-soil subgrade [4]. As the main stabilizer material of soft soil reinforcement, the demand for cement has increased dramatically with the development of infrastructure construction in recent years [5,6]. However, the production of cement places great pressure on the environment and energy [7,8]. It is estimated that the newly added stockpile of industrial waste in China exceeds 3 billion tons each year, and the accumulated stockpile is as high as 60 billion tons, while the comprehensive utilization rate is only 50–60%. As a result, a series of safe disposal and environmental pollution problems have occurred [9]. Replacing cement with industrial solid waste as the main cementitious material is an important way to promote environmental protection and waste residue recycling in the field of foundation treatment [10,11,12]. Slag and fly ash, with potential cementitious activities, are the most representative solid wastes available [13,14].

The complex salt environment has continued to have a negative impact on the performance of engineering materials [15,16,17]. Among them, sulfate erosion is one of the important factors that induces the failure of cement-stabilized soils [18]. Sulfate ions react with the hydration products of cement, causing the swelling, cracking, and spalling of stabilized soils, which in turn lead to the loss of strength and reduced durability of stabilized soils [19]. Yang et al. [20] found that the degree of deterioration of cement-stabilized soil is related to the dissolution of calcium ions in cement. Yu et al. [21] found that cement-stabilized soil was damaged after being immersed in a sulfate solution with a mass fraction of 2.5% for 90 d. A great deal of scattered ettringite was observed in the SEM images of the deteriorated area of the specimens, which would cause the expansion and cracking of the stabilized soil. To improve the strength and sulfate resistance of stabilized soils, scholars have tried to use industrial solid waste to partially or completely replace cement as stabilizers. Based on this, scholars from various countries have carried out many investigations of solid waste-based stabilized soil and have achieved outstanding results. Furlan et al. [22] and Horpibulsuk et al. [23] studied the strength and microstructure of clay stabilized by both fly ash and cement synergistically and found that adding fly ash could improve the strength and ductility of cement-stabilized soil. Pokharel et al. [24] confirmed that 10% calcium-based bentonite and 30% fly ash evidently facilitated an increase in the compressibility and strength of organic soils. Zhang et al. [25] found that the addition of slag can effectively improve the strength of cement-stabilized soil and has an excellent stabilization effect on metal elements. In terms of sulfate erosion, Xu et al. [26] used slag cement instead of ordinary Portland cement for coastal soft soil stabilization. It was found that slag-cement stabilized soil generates more hydration products, thereby limiting the influence of soluble salts in soft soil on cement-stabilized soil. Li et al. [27] studied the use of cement-silica fume-based stabilizers to stabilize soft soil under seawater erosion. The results show that the incorporation of ultra-fine silica fume increases the 90-day UCS of the stabilized soil by nearly 6.5% compared with the control group. However, the environmental pollution caused by cement utilization has not been completely solved. So far, the research work is mainly concentrated on understanding the inherent mechanisms of polymers through molecular dynamics simulations and micro-characterization technology [28,29,30]. There is still a lack of systematic research on the durability and microscopic mechanism of alkali-excited all-solid waste-based stabilized soils under sulfate erosion.

In this study, the appearance change, mass change, and UCS of cement and slag-fly ash-based geopolymer-stabilized soil under Na_2_SO_4_ erosion were investigated comparatively. The phase composition and microscopic characteristics of the stabilized soils were analyzed by XRD (X-ray diffraction) and an SEM (scanning electron microscope), and the sulfate resistance mechanism of the two types of stabilized soils was revealed. The research results can provide a reference for the technology of stabilizing soil with industrial waste in areas of sulfate erosion.

## 2. Materials and Test Program

### 2.1. Materials

The soft soil used in this experiment was taken from the Xiangjiang River Basin in China at a depth of 6–8 m. The physical and mechanical properties of the soil are shown in Table 1. The maximum particle size of the ground and sieved soil samples was less than 2 mm.

**Figure 1:**
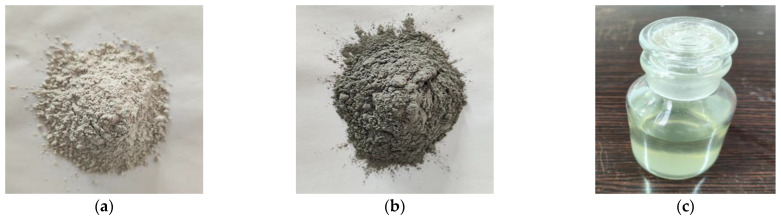
Pictures of raw materials: (**a**) Slag; (**b**) Fly ash; (**c**) Modified water-glass.

To investigate the effect of stabilizer types on the sulfate resistance of stabilized soils, two soil stabilizers (cement and geopolymer) were selected for this study. Geopolymer was prepared by using slag and fly ash (provided by Gongyi Longze Water Purification Material Co., Ltd., Zhengzhou, China) as raw materials, and modified water-glass as an alkali activator. Figure 1 shows the pictures of the raw materials. The chemical composition and physical indicators of P·O 42.5 cement, S95 slag, and Class F fly ash are shown in Table 2.

The alkali activator was prepared by mixing industrial water-glass and sodium hydroxide in a certain proportion (19.14 g sodium hydroxide per 100.0 g industrial water-glass), and its modulated modulus was 1.2. The modulus of industrial water-glass was 3.31, which was composed of 27.84% SiO_2_, 8.42% Na_2_O, and 63.74% H_2_O. Sodium hydroxide was of an analytical grade, and the content of NaOH exceeded 98%. The sulfate used in the erosion test was of analytical grade, and the content of Na_2_SO_4_ exceeded 98%.

### 2.2. Mix Proportions and Specimen Preparation

In this study, the moisture content of the reshaped soil was taken as 50.0%. According to the previous work, it was determined that the content of stabilizer (cement/geopolymer) is 25% of the soil mass. For geopolymer-stabilized soils, the effects of four slag-fly ash ratios (10:0, 9:1, 8:2 and 7:3) on the sulfate resistance of stabilized soils were investigated while keeping the alkali activator parameters constant. The specific mix proportions are shown in Table 3.

A cube mold with a side length of 70.7 mm was used for all immersion tests. According to the literature [31], after the fresh, stabilized soil mixture was molded, it was covered with plastic wrap until demolding after 24 h. The demolded specimens were cured under standard curing conditions for 28 days.

Subsequently, the specimens after standard curing were completely immersed in a 2.5% Na_2_SO_4_ solution, which was replaced every 7 days. The erosion ages were 0, 3, 7, 28, 60 and 90 d.

### 2.3. Test Methods

#### 2.3.1. Mass Change Rate

To accurately determine the mass change rate of the stabilized soil before and after immersion, the mass of three specimens was weighed for each erosion age, and the average value was taken. The calculation method of mass change rate (*ω*) is shown in Equation (1):

(1) $$\omega =\frac{m-m_{0}}{m_{0}}\times 100\%$$

where “*m*_0_” and “*m*” are the mass (unit: g) of the specimen before and after immersion, respectively.

#### 2.3.2. Unconfined Compressive Strength (UCS) and Strength Retention Coefficient

The loading rate of the UCS test was set to 1 mm/min, and the average strength of three parallel specimens was taken as the measured strength value [31]. The strength retention coefficient (*δ*_n_) is used to evaluate the degree of sulfate erosion, and the specific calculation is as shown in Equation (2).

(2) $$\delta_{n}=\frac{S_{n}}{S_{0}}\times 100\%$$

where “*S*_0_” and “*S*_n_” are the UCS values of the specimens eroded at 0 d and n d, respectively (unit: MPa).

#### 2.3.3. Microscopic Examination

The samples used in the microscopic test were taken from the inside of cube specimens. XRD experiment was performed using a x’pert Pro diffractometer (PANalytical B.V., Almelo, The Netherlands) with a scanning range of 5–90° and a scanning speed of 5°/min. SEM testing was performed using a Zeiss Sigma 300 instrument (Zeiss, Oberkochen, Germany), and the samples were sprayed with gold and vacuumed before testing.

## 3. Results and Discussion

### 3.1. Appearance Change

**Figure 2:**
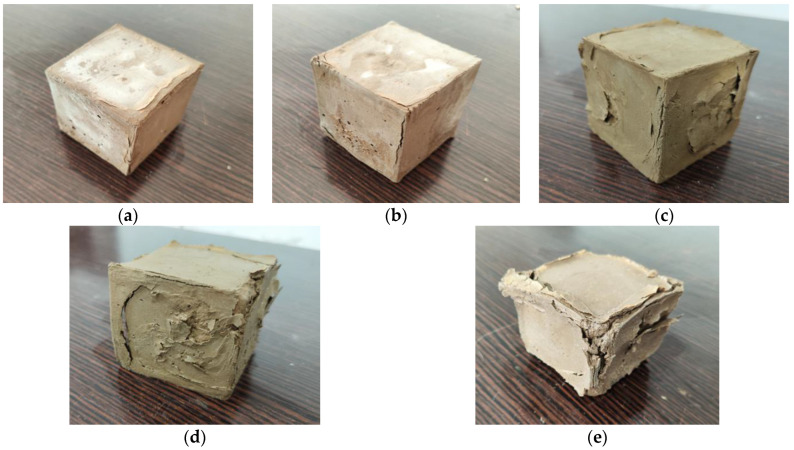
Appearance changes of cement-stabilized soil (C0 group) at different sulfate erosion ages: (**a**) 3 d; (**b**) 7 d; (**c**) 28 d; (**d**) 60 d; (**e**) 90 d.

Figure 2 shows the appearance changes of the cement-stabilized soil (C0 group) at different sulfate erosion ages. As evident in the figure, cement-stabilized soil is seriously deteriorated by Na_2_SO_4_ solution with time. When the erosion age is 3 d, the surface of the C0 specimen becomes rough, and it is also evident that the epidermis bulges and slight cracks appear at the angular edges of the specimen. The cracking phenomenon of the specimen becomes more pronounced with time, accompanied by local corrosion pits and small particles falling off. At 28 d, the further development of cracks causes the loose matrix structure of the specimen, resulting in more rapid subsequent sulfate erosion. When the erosion progresses to 90 d, the specimen shows severe cracking and spalling, and its integrity is damaged.

**Figure 3:**
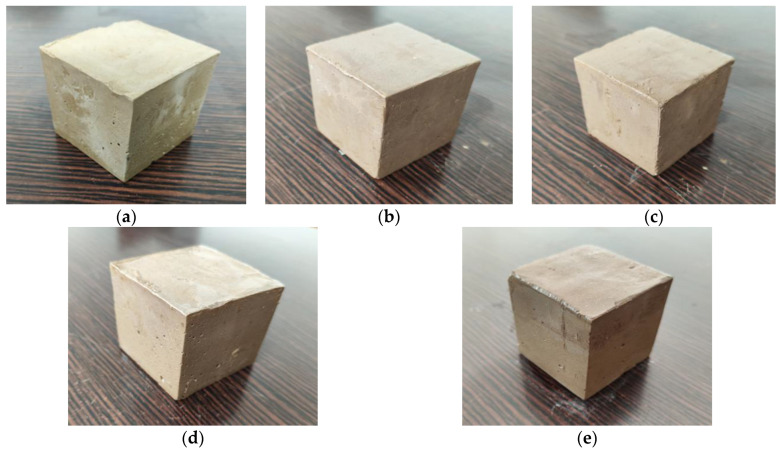
Appearance changes of geopolymer-stabilized soil (G-2 group) at different sulfate erosion ages: (**a**) 3 d; (**b**) 7 d; (**c**) 28 d; (**d**) 60 d; (**e**) 90 d.

Upon observing the appearance of the geopolymer-stabilized soil (G-2 group) at different sulfate erosion ages shown in Figure 3, it is evident that the appearances of the stabilized soil specimens are different due to the different stabilizer types. In the sulfate environment, the alkali-excited slag-fly ash-based geopolymer-stabilized soil always maintains a smooth surface within 7 d, and basically no cracks are formed. This shows that the rational utilization of industrial solid waste can effectively slow down or even resist the erosion of sulfate [22,26,32]. After 90 days of erosion, the matrix structure of the geopolymer-stabilized soil still maintains good integrity, and only the upper edge and part of the surface are damaged by a low degree of erosion. This indicates that the alkali-excited slag-fly ash stabilized soil has considerable resistance to sulfate erosion.

### 3.2. Mass Change

**Figure 4:**
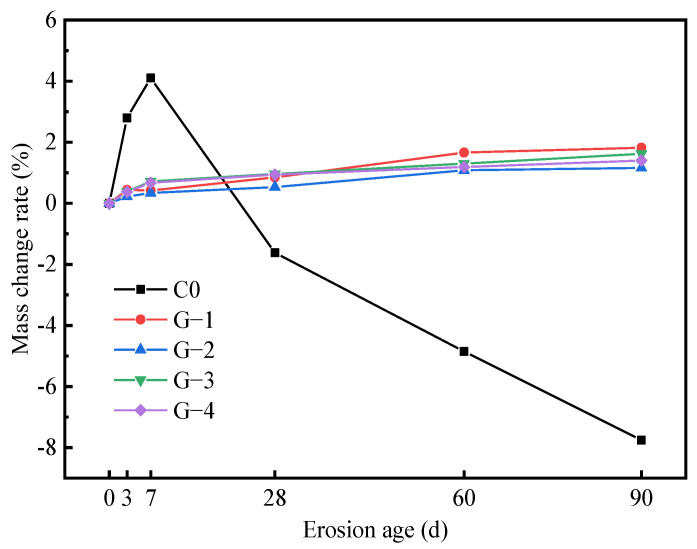
Mass change rate of stabilized soil immersed in sulfate erosion solution.

Figure 4 shows the mass change of the cement/geopolymer-stabilized soil with erosion age. It is evident that the mass change rates of cement-stabilized soil at 3 d, 7 d, 28 d, 60 d, and 90 d are +2.8%, +4.1%, −1.62%, −4.85%, and −7.76%, respectively. The results show that the mass of cement-stabilized soil exhibits two-stage variation characteristics within 90 days. Stage 1: In the first 7 days, the mass of the C0 specimen increases rapidly, which is mainly due to the rapid absorption of water in the Na_2_SO_4_ solution by the specimen through the defect site, accompanied by the production of ettringite, gypsum, and other expansive products. Stage 2: With the continuous progress of the sulfate erosion, the mass of the cement-stabilized soil decreases significantly. This is because the expansive products in the C0 specimen continue to generate, resulting in the damaging of their integrity and obvious spalling, as shown in Figure 2c–e.

As can be seen in Figure 4, different from the cement-stabilized soil, the mass of the geopolymer-stabilized soil changes slightly, and generally shows a steady increasing trend. This is because the mass stability of the specimen benefits from its integrity. The mass change of the specimen is controlled by the chemical reaction of the erosion process. The sulfate erosion of geopolymer-stabilized soil is accompanied by processes such as ettringite formation, gypsum crystallization, and the secondary hydration of mineral particles. These processes convert the water and related substances in the Na_2_SO_4_ solution into the product of the specimen, which in turn gradually increases in mass [26,27,33]. In contrast, G-2 with a slag-fly ash ratio of 9:1 has the best mass stability among the four geopolymer-stabilized soils. When the erosion ages are 3 d, 7 d, 28 d, 60 d, and 90 d, the mass change rates of the G-2 specimen are +0.22%, +0.34%, +0.53%, +1.08%, and +1.16%, respectively. This may be because the matrix structure of the G-2 specimen is densely developed, and its calcium hydroxide content is lower than that of the other groups. Therefore, the formation of expansive products and the propagation of cracks are suppressed, so that a substantial increase in mass due to water absorption by the cracks will not be caused.

### 3.3. Strength Development

**Figure 5:**
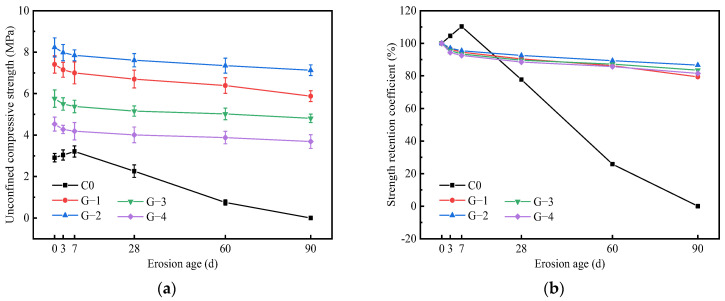
Strength development of stabilized soil immersed in sulfate erosion solution: (**a**) UCS value; (**b**) strength retention coefficient.

Figure 5 shows the change of the UCS value of cement-stabilized soil with erosion age under a sulfate environment. In general, the strength of cement-stabilized soil increases gradually and then decreases sharply with age. For the cement-stabilized soil immersed for 0 d (standard curing 28 d), its UCS value is 2.91 MPa. As observed, the short-term immersion (within 7 days) is beneficial to increasing the UCS of cement-stabilized soil. Specifically, the 3 d and 7 d UCS values of the cement-stabilized soil are 3.04 and 3.21 MPa, respectively. This slow increase in strength is the result of the positive effect of the newly formed hydration products and the reduction in the strength of the cement-stabilized soil caused by erosive ions. The mechanism of the positive effect can be explained as the chemical reaction between some cementitious substances such as C-S-H and Ca(OH)_2_ in the cement-stabilized soil and sulfates to form expansive substances such as ettringite (AFt) and gypsum, which then fill the pores in the stabilized soil, thereby improving the strength of the stabilized soil [18,19,34]. However, with the deepening of the sulfate erosion, the large number of expansive substances formed increases the internal stress in the stabilized soil, resulting in cracks inside the specimen. Macroscopically, the strength loss of the stabilized soil is significant. The UCS of cement-stabilized soil decreases from 2.26 MPa at 28 d to 0.75 MPa at 60 d. Finally, the strength is completely lost at 90 d. This is manifested as the disintegration of the matrix structure’s appearance, as shown in Figure 2e.

Observing the changes in the UCS values and strength retention coefficients of geopolymer-stabilized soil in Figure 5a,b, it is evident that the UCS values of the G-1, G-2, G-3, and G-4 that have not been immersed are 7.41, 8.23, 5.76, and 4.53 MPa, respectively. In contrast, the G-2 with a slag-fly ash ratio of 9:1 after a standard curing for 28 d has the most complete strength development and the densest matrix structure [35]. It is also clear that the strength development law of the specimens under the Na_2_SO_4_ solution environment is basically similar. Specifically, the UCS of the four geopolymer-stabilized soils decreases gradually with the erosion age, and the decline rate of their UCS slows down significantly after 7 d. Based on this, the erosion-deterioration process of sulfate on geopolymer-stabilized soil is divided into two stages: an initial acceleration period and a stable period. The initial acceleration period mainly involves the erosion-deterioration behavior of ${SO}_{4}^{2-}$ entering the stabilized soil from the pores. ${SO}_{4}^{2-}$ ions react with the partial hydration products of geopolymers to form expansive crystals, which fill the capillary pores and hinder the migration of erosive ions. After that, the erosion process enters a stable period. Obviously, different slag-fly ash ratios cause differences in the sulfate erosion resistance of geopolymer-stabilized soil. Moreover, the early integrity and initial strength of stabilized soil are important factors that affect its sulfate resistance. Comparing the strength development of the four geopolymer-stabilized soils, it is evident that G-2 has the most excellent sulfate resistance. When the erosion age is 90 d, the UCS value of G-2 is 7.13 MPa, and its strength retention coefficient is 86.6%.

### 3.4. Phase Analysis

**Figure 6:**
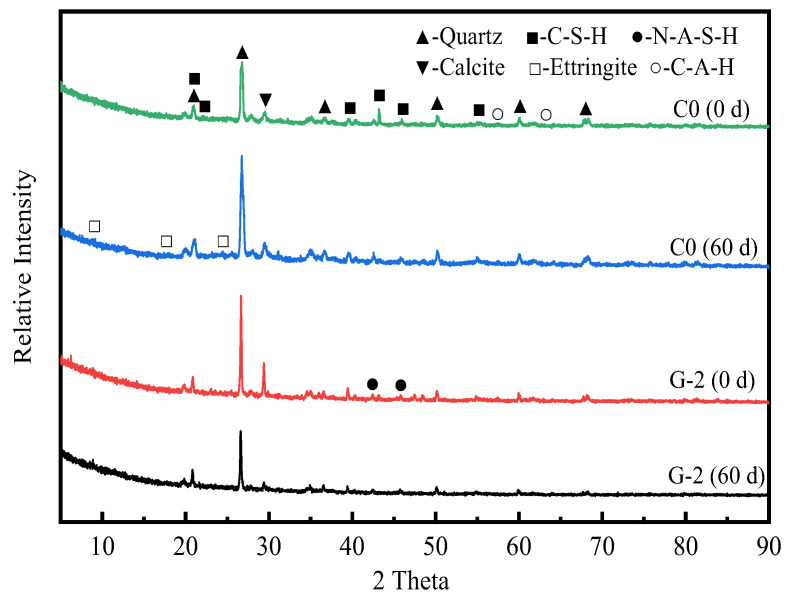
XRD pattern of stabilized soil immersed in sulfate erosion solution.

The sulfate erosion resistance of different stabilized soils is obviously different, which may be due to the different types and structures of the generated products. The phase evolution of the geopolymer-stabilized soil under an Na_2_SO_4_ erosion environment was determined by XRD test, and compared with the cement-stabilized soil at the corresponding age. The obtained XRD pattern is shown in Figure 6.

As observed, the quartz phase in the stabilized soil remains essentially unchanged after immersion in the Na_2_SO_4_ solution, which is related to its inert nature. Comparing the XRD patterns of C0 (0 d) and C0 (60 d), it is evident that the main products of the cement-stabilized soil are C-S-H gel and a small amount of C-A-H. After immersing it in Na_2_SO_4_ solution for 60 d, the AFt diffraction peaks in the cement-stabilized soil are significantly enhanced. However, the erosion products dominated by AFt accelerate the structural failure of stabilized soils. Macroscopically, this is manifested as the expansive cracking of the stabilized soil, which is consistent with the appearance change of the cement-stabilized soil (as shown in Figure 2).

The different slag-fly ash ratios of geopolymers will inevitably lead to differences in the content of their reaction products but will not affect their phase composition. This section focuses on the G-2 group of geopolymer-stabilized soil, which has excellent macroscopic properties. From the XRD pattern of G-2 (0 d), no obvious AFt (expansive product) is observed. As for G-2 (60 d), there are several weak AFt diffraction peaks. It is evident from Table 2 that the CaO content of slag and fly ash is lower than that of cement; thus, they lack the ion concentration required to generate AFt [36]. The XRD results show that the main product of the geopolymer-stabilized soil is hydrated sodium aluminosilicate (N-A-S-H) gel, which has a very stable three-dimensional network structure. This is also the main reason why the sulfate resistance of geopolymer-stabilized soil is more excellent than that of cement-stabilized soil.

### 3.5. Microstructure

**Figure 7:**
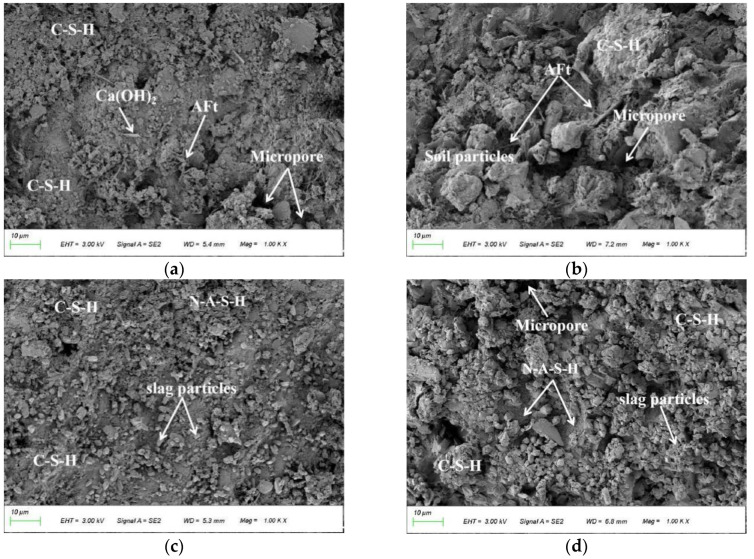
SEM images of stabilized soil immersed in sulfate erosion solution: (**a**) C0 (0 d); (**b**) C0 (60 d); (**c**) G-2 (0 d); (**d**) G-2 (60 d).

Figure 7 shows the SEM images of the cement-stabilized soil/geopolymer-stabilized soil fully immersed in Na_2_SO_4_ solution. In Figure 7a, the microstructure of the unsoaked cement-stabilized soil is mainly composed of a small amount of randomly distributed AFt crystals, C-S-H gels, and flaky Ca(OH)_2_ crystals. As shown in Figure 7b, the number of AFt crystals in the cement-stabilized soil increases sharply after sulfate erosion for 60 d. In addition, the AFt crystals gradually change from being acicular to rod-like, resulting in an increase in the internal stress of the stabilized soil [18]. Meanwhile, the cohesion between the cement and soil particles is seriously weakened by the intrusion of sulfate, resulting in exposed soil particles, and increasing particle gaps [19,21]. As a result, the strength of the cement-stabilized soil seriously deteriorates.

Compared with the cement-stabilized soil under the same conditions, the geopolymer-stabilized soil has a denser initial microstructure (Figure 7c). As observed in Figure 7d, the geopolymer-stabilized soil particles are tightly packed and encapsulated by the polymerization products. In addition, almost no AFt crystals (expansive product) were formed in the geopolymer-stabilized soil. Moreover, stable network structure products were uniformly distributed on the surface of the stabilized soil particles. When combined with the XRD results, they were determined to be N-A-S-H gels (typical polymerization products) [31,32]. The N-A-S-H gel contributes to enhancing the bonding strength between soil particles and preventing the intrusion of free sulfate ions into the stabilized soil [26].

## 4. Conclusions

The sulfate erosion resistance of cement-stabilized soil and slag-fly ash-based geopolymer-stabilized soil was comparatively studied based on the tests of appearance change, mass change, strength development, and microscopic examination. The following valuable conclusions have been drawn:

(1) The type of stabilizer is a key factor affecting the sulfate resistance of stabilized soil. Sulfates severely deteriorate cement-stabilized soil with time, even damaging its integrity. The mass of cement-stabilized soil shows a two-stage variation characteristic of an initial increase and then a decrease within 90 days. Specifically, the mass change rates of the cement-stabilized soil at 3 d, 7 d, 28 d, 60 d, and 90 d were +2.8%, +4.1%, −1.62%, −4.85%, and −7.76%, respectively. Geopolymer-stabilized soils show a slight erosion-deterioration phenomenon in a sulfate environment, and their masses generally show a slightly increasing trend (less than 1.82%).
(2) The UCS of cement-stabilized soil increases gradually with its immersion time, and then it decreases sharply. The cement-stabilized soil obtains a maximum strength of 3.21 MPa at 7 d, while its strength is completely lost when immersed for 90 d. The slag-fly ash ratio has little effect on the strength development law of geopolymer-stabilized soil under a Na_2_SO_4_ solution environment, but it is an important factor affecting the sulfate resistance of stabilized soil. In contrast, G-2 with a slag-fly ash ratio of 9:1 has the most excellent sulfate resistance. When the erosion age is 90 d, the UCS value of G-2 is 7.13 MPa, and its strength retention coefficient is 86.6%.
(3) After immersion in Na_2_SO_4_ solution for 60 d, a large amount of AFt (expansive crystal) is formed in the cement-stabilized soil. The expansion stress generated by AFt damages the soil structure, resulting in the gradual deterioration of the strength of the cement-stabilized soil until it is completely lost. The N-A-S-H gel in the geopolymer-stabilized soil enhances the bonding strength between soil particles, and its stable microstructure retards the intrusion of free sulfate ions into the stabilized soil.

## Figures and Tables

**Table 1:** Physical and mechanical properties of soil.

Natural Moisture Content/%	Wet Density/g·cm^−3^	Void Ratio	Liquid Limit/%	Plasticity Index	Cohesion/kPa	Internal Friction Angle/°	Compression Modulus/MPa
49.8	1.71	1.196	33.2	17	13.5	2.5	3.37

**Table 2:** Chemical composition and physical indicators of raw materials (wt%).

Raw Materials	CaO	SiO_2_	Al_2_O_3_	MgO	Fe_2_O_3_	SO_3_	Others	LOI	Specific Surface areas/m^2^·kg^−1^
Cement	56.43	19.55	5.63	3.54	2.96	2.83	9.06	2.08	342
Slag	34.00	34.50	17.70	6.01	1.03	1.64	5.12	1.83	505
Fly ash	3.23	49.04	27.4	0.86	1.53	1.15	16.79	2.36	935

**Table 3:** Mix proportions.

Label	Cement Content/%	Geopolymer Content/%	Alkali Activator		Slag: Fly Ash	*w*/*b*
			Modulus	Content/%		
C0	25	-	-	-	-	0.4
G-1	-	25	1.2	30	10:0	0.4
G-2	-	25	1.2	30	9:1	0.4
G-3	-	25	1.2	30	8:2	0.4
G-4	-	25	1.2	30	7:3	0.4

## Data Availability

Not applicable.
